# Hypoglycemia: Elucidating its circadian propensity and recovery time based on clinical parameters

**DOI:** 10.22088/cjim.13.1.29

**Published:** 2022

**Authors:** Ravi Kant, Poonam Yadav, Madhuri Pratti, Shruti Barnwal

**Affiliations:** 1 Department of Medicine, All India Institute of Medical Sciences, Rishikesh, India; 2 Center of Excellence in Nursing Education and Research, All India Institute of Medical Sciences, Rishikesh, India; 3 Department of Dermatology, Govt. Doon Medical College, Dehradun, India

**Keywords:** Anemia, Body mass index, Comorbidity, Diabetes mellitus, Hypoglycemia, Hypoglycemic episode, Insulin, Retinopathy.

## Abstract

**Background:**

Hypoglycemia is frequently associated with insulin therapy in diabetic patients; it leads to many short and long-term complications and even death if not addressed in time. This study was undertaken to observe the circadian propensity of hypoglycemia and its recovery time based on type 2 diabetes mellitus patients’ clinical parameters.

**Methods:**

We included type 2 diabetes mellitus (DM) hospitalized patients with the exclusion of patients suffering from critical illness. Data were collected for a period of three months (September to November 2019).

**Results:**

A total of 120 patients were included, comprising 60% males and 40% females. Approximately 55% of patients had hypoglycemic episodes at around 12 am– 6 am. The most common comorbidity present in these patients was hypertension (43.3%, P=0.931). Anemia (OR-3.765, CI-1.350-5.500, P=0.011), retinopathy (OR 6.066, CI-2.031-8.113, P=0.001), and duration of DM (OR-6.266, CI-2.209-7.774, P=0.001) were significantly associated with the recovery time of hypoglycemia, around 50±14.14min in the elderly population of age 60-70. People with BMI 22.5- 27.5 Kg/m^2 ^took around 45.66 ± 19.37 min to recover after treatment.

**Conclusion:**

Time taken to recover from hypoglycemic episodes vary with age and BMI**. **Associated comorbidities such as anemia, retinopathy, and DM duration had a significant bearing on the time taken to recover from hypoglycemia. Recovery time was directly proportional to BMI, a new finding that needs further molecular level evaluation. Circadian propensity of hypoglycemia in these patients have been identified in the early morning hours of the day.

Diabetes mellitus (DM) is a group of metabolic diseases that share a hyperglycemia phenotype. According to ADA, diabetes mellitus is diagnosed as symptoms of diabetes with random blood glucose concentration ≥11.1 mmol/L (200 mg/dL) (or), fasting plasma glucose ≥7.0 mmol/L (126 mg/dl), HbA1c ≥ 6.5% (or), 2-h plasma glucose ≥11.1 mmol/L (200 mg/dL) during an oral glucose tolerance test (1). It is the leading cause of comorbidities like end-stage renal disease (ESRD), non-traumatic lower extremity amputations, and blindness. It also predisposes to cardiovascular diseases (2). The prevalence of DM is increasing, and as per IDF projections, 642 million individuals will have diabetes by 2040 (3). Increasing prevalence and incidence worldwide, diabetes-related complications, both microvascular and macro-vascular, continues to be a leading cause of morbidity and mortality in the near future (2).

Glycemic control is the main target of oral medications or insulin injections to reduce the burden of DM-related complications (4, 5). Major landmark studies like Diabetes Control and Complications Trial (DCCT) performed in patients of type 1 diabetes mellitus and the United Kingdom Prospective Diabetes Study (UKPDS) in type 2 diabetic patients showed that strict glycemic control has a beneficial effect on microvascular complications (6). Hypoglycemia is not only a barrier in achieving optimum control, but it also had many adverse cardiovascular effects. Hypoglycemia is a common and avoidable complication of diabetes treatment. Iatrogenic hypoglycemia is a clinical condition arising due to absolute or relative insulin excess and compromised glucose counter-regulation in type1 and advanced type 2 diabetes (7). The goal of glycemic management is lifetime euglycemia without hypoglycemia that undoubtedly requires glucose-regulated insulin replacement or secretion. ^[^^8^^] ^Thus iatrogenic hypoglycemia is a confounder for attaining euglycemia, and studying the characteristic features and factors leading to hypoglycemic episodes is an important area of research (9). Data showed that circadian propensity develops cardiovascular risk even in healthy adults due to disruption in the biological clock (10, 11). Evidence from studies depicted its clinical significance in diabetes patients as it affects glucose tolerance through different mechanisms and regulates glucose metabolism by lowering insulin sensitivity, not by affecting β-cell function (12, 13). This study aimed to see the circadian propensity of hypoglycemia and its recovery time based on clinical parameters in type 2 diabetes patients on insulin therapy. 

## Methods

**Study Design: **It was a hospital-based prospective observational study that included diabetic patients on insulin therapy with documented hypoglycemia. 

**Sample size: **Raosoft electronic software was used to calculate the sample size (based on the following factors: 95% confidence interval, 5% acceptable margin of error, and population size of 170 diabetic patients with an estimated prevalence of hypoglycemia = 50%). The calculated sample size was 119 patients. 

**Sampling technique: **Recruitment of 120 participants was done with the purposive sampling technique. 

**Eligibility criteria: **Type 2 diabetic patients on basal-bolus insulin regimen with varying insulin doses and documented hypoglycemia episodes were included in the study, though all critically ill and post-operative patients were excluded. 

**Study Setting: **Data were collected for three months (September 2019- November 2019) from the Endocrine and General Medicine ward at a tertiary care hospital, India.

**Steps of data collection: **All the participants were informed, and written consent was taken when enrolled. Data were collected from the patients of type 2 diabetes mellitus on insulin therapy and those who had acquired hypoglycemia during hospitalization in the Endocrine ward and General Medicine ward at a tertiary care hospital. All patients were on basal-bolus insulin therapy (three short-acting and one long-acting insulin). Since these patients were admitted, the time of insulin injections was fixed and relatively the same as the meals provided by the hospital at a fixed time. A case reporting form was used to collect data. Case sheets of patients were observed for the presence of comorbidities (such as hypertension, anemia, diabetic nephropathy, retinopathy, coronary artery disease, and duration of disease) at the time of admission to the hospital.

**Diagnostic criteria**

**Hypoglycemia:**

Hypoglycemia is defined by

- Plasma glucose values (biochemically)
- Symptoms (clinically)
- Time of occurrence (day/night) (7)

ADA has classified hypoglycemia as follows:

Level 1: -Glucose <70mg/dl and > 54mg/dl

Level 2: - Glucose <54mg/dl

Level 3: - Severe event characterized by altered mental & or physical status

**Hypertension: **Hypertension is diagnosed when systolic blood pressure (SBP) is ≥140 mm Hg and /or diastolic blood pressure (DBP) is ≥90 mm Hg following repeated examination (14).

**Anemia: **According to World Health Organization, anemia’s diagnostic criteria are a hemoglobin value of less than 13 g/dL in male and 11 female adults (15).

**Diabetic Nephropathy: **Early stages of nephropathy was identified with microalbuminuria with a spot urine albumin/creatinine ratio. In six months, positive results for two tests out of three tests with a value of 30 to 300 mg of albumin/g of creatinine is the diagnostic criteria for diabetic nephropathy (16).

**Diabetic neuropathy: **A physician diagnosed diabetic neuropathy after assessing the clinical sign and symptoms, nerve conduction test, and quantitative sensory testing of diabetic patients (17).

**Coronary Artery Disease: **Coronary artery disease’s diagnosis is based on precordial pain suggestive of cardiac in origin along with baseline electrocardiography (ECG), Echocardiography (including stress echocardiography), Coronary angiography (18). 

**Diabetic Retinopathy: **Ophthalmoscopy with or without dilated pupil is the standard procedure in the screening for diabetic retinopathy by a trained ophthalmologist, in which detection of microaneurysms in the posterior pole is the earliest clinical sign (19).

**Statistical analysis: **Statistical analysis was performed using software (SPSS 23.0 Version). The chi-square and multiple linear regression analysis (p-value significant as < 0.05) was applied to measure the effect of various factors on hypoglycemia recovery time.

**Ethics statement: **The protocol was reviewed and approved by the Institutional Ethics Committee (IEC) (01/IEC/IM/NF/2018). At the enrollment phase, written and informed consent was obtained from all participants.

## Results

**fig. 1:**
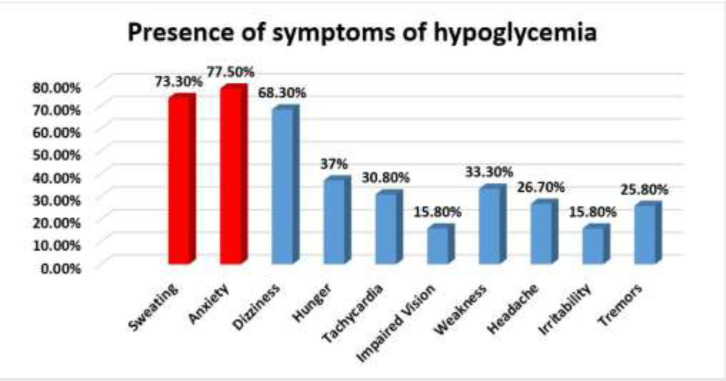
Symptoms of hypoglycemia with insulin therapy in diabetes patients

A total of 120 patients were enrolled, comprising 72 (60%) males and 48 (40%) females. 23 (19.2%) patients had a family history of diabetes.70 (58.3%) patients were non-vegetarian. Other demographic characteristics have been shown in table 1. Patients’ mean age was 56.5±8.4 years, with a mean duration of diabetes mellitus of 7.84±4.128 years. The most common comorbidity associated with these patients was hypertension (43.3%), followed by anemia (42.5), nephropathy (25.8 %), neuropathy (20.8%), coronary artery disease (20%), retinopathy (18.33%). The most common comorbidity present in these patients was hypertension (43.3%, P=0.931). Anemia (OR-3.765, CI-1.350-5.500, P=0.011), retinopathy (OR 6.066, CI-2.031-8.113, P=0.001), and duration of DM (OR-6.266, CI-2.209-7.774, P=0.001) were significantly associated with the recovery time of hypoglycemia. (Multiple linear regression, p<0.05) (table 2). The most common symptoms of hypoglycemia observed in these patients were anxiety (77.50%), followed by sweating (73.30%), dizziness (68.30%), hunger (37%), tachycardia (30.80%), weakness (30.30%), headache (26.70%), tremors (25.80%), impaired vision (15.80%), and irritability (15.8%) (figure 1).

**Figure 2:**
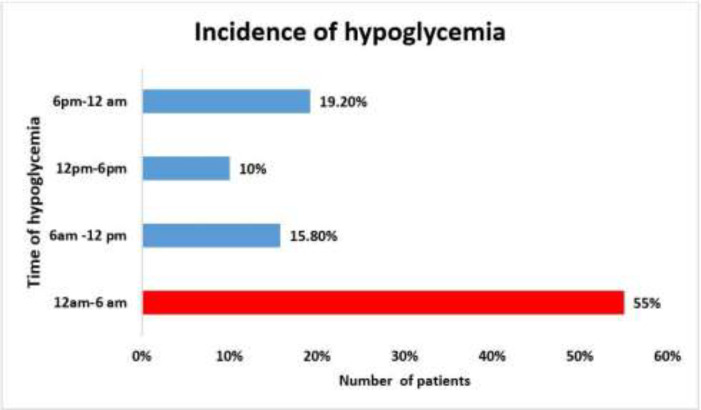
Incidence of hypoglycemia concerning time periods

About 55% of patients had hypoglycemic episodes at around 12 am – 6 am and 19.2% between 6 pm to 12 am, 15.8% of patients experienced it between 6 am -12noon and 10% between 12noon – 6 pm (figure 2).

**Table 1:** Demographic characteristics of participants

**Variables **	**Frequency %**	**P-Value**
**Age **		0.016
<40 Yrs.	23 (19.16)	
40-50 Yrs.	36 (30)	
50-60 Yrs.	52 (43.33)	
60-70 Yrs.	7 (5.83)	
>70 Yrs.	2 (1.66)	
**Gender**		0.363
Male	72 (60)	
Female	48 (40)	
**Education qualifications **		0.103
Un-Educated	39 (32.5)	
Intermediate	35 (29.1)	
secondary	34 (28.4)	
Graduate and above	12 (10)	
**Marital Status**		0.217
Married	92 (76.6)	
Single	28 (23.3)	
**Family History of diabetes**		0.387
Yes	23 (19.2)	
No	97 (80.8)	
**Dietary habits**		
Vegetarian	50 (41.7)	
Non-Vegetarian	70 (58.3)	
**Treatment**		0.189
Insulin only	48 (40)	
Oral+ Insulin (Combined)	72 (60)	
**BMI**		0.048*
<17 Kg/m^2^	12 (10)	
17-22.5 Kg/m^2^	77 (64.10)	
22.5- 27.5 Kg/m^2^	30 (25)	
>27.5 Kg/m^2^	1 (0.83)	
**Duration of DM**		0.000*
1-5 Yrs.	41 (34.2)	
5-10 Yrs.	51(42.5)	
10-15 Yrs.	21 (17.5)	
15-20 Yrs.	7 (5.8)	

Chi-square test, p value significant >0.05*

BMI- body mass index, DM- diabetes mellitus

The time duration required for patients to recover from hypoglycemic episodes varied with age and BMI. The majority of patients (31%) recovered from hypoglycemia in 15-30 min, and 27.45% of patients followed by 45-60 min. Subsequent subgroup analysis of those recovering from hypoglycemia had male predominance (39/15, M: F), and 35 (29.16%) patients had BMI between 17-22.5 Kg/m^2^. Time taken to recover from hypoglycemia is more in the elderly population of 60-70 years of age. It was around 50±14.14 min, compared to the young population. People with BMI 22.5- 27.5 Kg/m^2^ took an average of 45.66±19.37 min to recover after treatment, which is more than the time taken by patients with lower BMI. One patient with BMI >27.5 Kg/m^2^ took 90 min to recover from hypoglycemia. Female patients took a long time (43.17±18.94) to recover as compared to male patients (39±14.74) (table 3). 

**Table 2:** Relationship of variables in relation to the recovery time of Hypoglycaemia n= 120

**Variables** **Associated Comorbidities **	**Frequency (%)**	**Exp. (B)** **OR**	**P-Value**	**CI**
HTN	52 (43.3)	1.042	0.931	0.410-2.650
Anaemia	51 (42.5)	3.765	0.011*	1.350-5.500
Nephropathy	31 (25.8)	0.820	0.705	0.295-2.284
Neuropathy	26 (20.8)	0.969	0.954	0.330-2.844
CAD	24 (20)	1.046	0.940	0.326-3.357
Retinopathy	22 (18.33)	6.066	0.001**	2.031-8.113
Duration of DM	-	6.266	0.001**	2.209-7.774

Multiple Linear regression analysis, p value significant <0.05*, < 0.01**

HTN-hypertension, CAD- coronary artery disease, DM- diabetes mellitus,

CI-confidence interval, OR- Odds Ratio.

**Table 3:** Details of recovery periods of hypoglycaemia

	**15-30 min**	**31-45 min**	**46-60 min**	**61-75 min**	**76-90 min**	**Mean **± **SD**
**Age**						
<40 Yrs.	17 (14.16)	5 (4.16)	1 (0.83)	0 (0)	0 (0)	31.30 ± 9.91
40-50 Yrs.	18 (15)	6 (5)	9 (7.5)	0 (0)	3 (2.5)	41.94 ±21.39
50-60 Yrs.	18 (15)	15 (12.5)	16 (13.3)	2 (1.66)	1 (0.83)	44.90 ±16.81
60-70 Yrs.	1 (0.83)	1 (0.83)	5 (4.16)	0 (0)	0 (0)	50 ± 14.14
>70 Yrs.	0 (0)	0 (0)	2 (1.66)	0 (0)	0 (0)	60 ± 0.0
BMI*						
<17 Kg/m^2^	8 (6.6)	2 (1.66)	2 (1.66)	0 (0)	0 (0)	32.91 ±15.87
17-22.5 Kg/m^2^	35 (29.16)	19 (15.83)	21 (17.5)	1 (0.83)	1 (0.83)	41.29 ±16.45
22.5- 27.5 Kg/m^2^	11 (9.16)	6 (5)	10 (8.3)	1 (0.83)	2 (1.66)	45.66 ±19.37
>27.5 Kg/m^2^	0 (0)	0 (0)	0 (0)	0 (0)	1 (0.83)	90 min
**Gender**						
Male	39 (32.5)	16 (13.3)	24 (20)	2 (1.66)	4 (3.33)	39 ± 14.74
Female	15 (12.5)	11 (9.16)	9 (7.5)	0 (0)	0 (0)	43.17 ±18.94

*BMI- body mass index

**Table 4:** Describing normal glycemic thresholds

**Glucose level**	**Response**	**Effects**	**Role in the correction of hypoglycemia**
80-85 mg/dl	Decreased insulin	Increased lipolysis, increase fatty acid	1^st^ defense against hypoglycemia
65-70 mg/dl	Increased Glucagon	Increased glucose production	2^nd^ defense against hypoglycemia
65-70 mg/dl	Increased Epinephrine	Increased lipolysis, Increased free fatty acid	3^rd^ defense, critical when glucagon is deficient
65-70 mg/dl	Increased Cortisol and growth hormone	Increased glucose production Decreased glucose clearance	Involved in defense against prolonged hypoglycemia
50-55 mg/dl	Symptoms	Recognition of hypoglycemia	Behavioral defense (food ingestion)
< 50 mg/dl	Decreased cognition	-	Compromises behavioral defense against hypoglycemia

## Discussion

In inpatient diabetes management, a pendulum has swung from achieving intensive glucose control towards more moderate and individualized glycemic targets. Many studies showed that recurrent hypoglycemia leads to failure of the counter-regulatory mechanism, but also mortality is high among patients who experienced hypoglycemic episodes than those who did not (20, 21). Despite strong evidence of the likely benefit of strict glycemic control, those trying to decrease the risk of microvascular complications through intensive glycemic control inevitably face a 3-fold increased risk of severe hypoglycemia (22). Hypoglycemia is commonly caused by drugs that are used to treat diabetes mellitus (23). The present study described hypoglycemia episodes and the effect of various factors leading to hypoglycemia and recovery.

**Clinical symptoms of patients with hypoglycemia: **The most common symptoms observed in patients were anxiety, sweating, dizziness, hunger, tachycardia, weakness, headache, tremors, impaired vision, and irritability. A recently conducted cross-sectional study on 390 diabetic patients established the high prevalence (57.44%) of hypoglycemia in India’s rural region, and the most typical reported symptom of hypoglycemia was dizziness in approx. 72.3% of patients (24). AlKhaldi YM et al. also performed a prospective study and observed sweating (49%), palpitation (48%), blurring of vision (31%), dizziness (9%), and headache (7%) as the most common symptoms during hypoglycemic episodes in diabetic patients (25). A retrospective study involving 1566 patients identified hypoglycemia with low conscious levels (60.63%), giddiness (22%), sweating (3.54%), and palpitations, 1.06% with motor deficits, and 11.35% had nonspecific symptoms (26). Even Tsujimoto T et al., in an observational study, found that the prior use of beta-blockers may prevent adverse events like hypertension and hypokalemia during severe hypoglycemia in diabetic patients (27). There are complex control mechanisms that maintain plasma glucose homeostasis (table 4).

Thus, in normal people, glucose-sensing neurons located in the brain and other regions detect hypoglycemia and initiate hormonal and neural responses through complicated defense mechanisms (28). However, in patients receiving insulin and insulin secretagogues, the counterregulatory mechanisms are impaired. Repeated episodes of hypoglycemia lead to impaired reduced counter-regulatory response and hypoglycemic awareness. Potential mechanisms that can lead to impaired glucose regulation are upregulation of glucose transport to the brain, utilization of alternate fuels to maintain energy metabolism during hypoglycemia, increased glycogen storage, altered hypothalamic neuro signaling, and increased cerebral oxidative stress (29).

**Timings of hypoglycemia: **In the present study, about 55% of patients had hypoglycemic episodes around midnight to 6 am. Consistently, a retrospective hospital-based study noted most hypoglycemic events between midnight to 6 am in diabetic patients (30). Cun-mei Yang et al. also observed that among 1374 cases, 2.40% of patients developed hypoglycemia between midnight to 2 am, 2% during 2 am -4 am, and 1.50 % developed during 10 am to 12 pm. However, severe hypoglycemic episodes were found from midnight to 2 am. ^[31] ^In contrast to findings, Ruan Y et al. conducted a retrospective observational study in 17,658 diabetes patients to explore hypoglycemia distribution. They found higher hypoglycemic events just before mealtimes, approximately three hours after lunch and dinner, and breakfast peaks at 11 am, 4 pm, and midnight. The highest number of hypoglycemia episodes were seen between 1 am, and 5 am in diabetic patients (32). AlKhaldi YM et al. also observed that 36% and 31% of diabetic patients experienced hypoglycemia in the morning and evening time respectively, and < 25% experienced hypoglycemia at night time, but time distribution was not specified in this study (25).

**Recovery of hypoglycemia: **In our study, the time required for patients to recover from hypoglycemic episodes varied with age and BMI. Older people and those with higher BMI (>22.5 Kg/m2) took more time to recover from hypoglycemia (45.66 min). In contrast to findings, a study conducted in Saudi Arabia with 378 participants found that patients of younger age group, insulin-dependent DM with long disease duration were risk factors for hypoglycemia in the diabetic population (25). A prospective study observed that BMI is also associated with hypoglycemic events in a young diabetic population 30–49 years with comorbidities (33). Vilovic M et al. found that hypoglycemia patients had a long duration of DM and their BMI values were 26.38 kg/m^2 ^(35). As we have not found enough literature regarding associated factors with the time required to recover from hypoglycemia, our study’s findings contribute to its significance in this perspective.

**Other aspects: **In the present study, hypertension was the most common comorbidity associated with these patients. However, other comorbidities such as anemia, nephropathy, neuropathy, coronary artery disease, and retinopathy were also present in these patients. Nevertheless, anemia, retinopathy, and DM duration were significantly associated with time taken to recover from hypoglycemia. In previous studies, disorders like – critical organ failure, sepsis, non-β cell tumors, and insulinoma are also risk factors for hypoglycemia (35-37). Kim HM et al. also observed that hypertension (80.8%) was the most prevalent comorbidity, followed by dyslipidemia and ischemic heart disease among diabetic patients experiencing hypoglycemia. Comorbidities such as chronic kidney disease and dementia were also associated with the risk of hypoglycemia and hospitalization amongst older patients with diabetes (38). Economically, hypoglycemia burdens the healthcare system and adversely affects workplace productivity, particularly after a nocturnal event. Selecting or modifying therapy to reduce hypoglycemia can take one of the variables of diabetes management and turn it into somewhat more of a constant, minimizing hypoglycemia risk (39, 40).

Knowing the circadian propensity of hypoglycemia and characteristics of people suffering from hypoglycemic episodes often increase the insights into their inter-relation with different characteristics and help them be extra cautious while prescribing the medications. Thus, in this study, many characteristics of diabetic people have been evaluated concerning hypoglycemia; however, it is a single hospital-based study with a limited sample size. Further extensive studies might be required at the molecular level to establish their relationship.

In conclusion, despite the known benefits of strict glycemic control on diabetes-related complications, hypoglycemia often became a barrier for achieving the goals, as recurrent episodes of hypoglycemia lead to increased cardiovascular risk and altered counter-regulatory mechanisms. The study concludes that associated comorbidities such as anemia, retinopathy, and DM duration had a significant bearing on the time taken to recover from hypoglycemia. Time taken to recover from hypoglycemic episodes also vary with age and BMI. 

Apart from elderly patients being at risk of developing complications, the time taken to recover is also more in these patients. People with high BMI took a long time than those with lower BMI, which is a new finding that needs further molecular level evaluation. Hypoglycemic episodes were more common from midnight to the morning at 6 am. Circadian propensity of hypoglycemia in these patients have been identified in early morning hours of the day.

## Funding:

No funding assistance granted.

## Conflict of interest:

None declared.

## References

[1] American Diabetes Association (2015). Classification and Diagnosis of Diabetes. Diabetes Care.

[2] Harding JL, Pavkov ME, Magliano DJ, Shaw JE, Gregg EW (2018). Global trends in diabetes complications: a review of current evidence. Diabetologia.

[3] International Diabetes Federation (2019). IDF Diabetes Atlas.

[4] Chen SY, Hsu HC, Wang RH, Lee YJ, Hsieh CH (2018). Glycemic control in insulin-treated patients with type 2 diabetes: empowerment perceptions and diabetes distress as important determinants. Biol Res Nurs.

[5] Cryer PE, Irene E, Karl MM (2007). Insulin therapy, and hypoglycaemia in type 2 diabetes mellitus. Insulin.

[6] King P, Peacock I, Donnelly R (2001). The UK Prospective Diabetes Study (UKPDS): clinical and therapeutic implications for type 2 diabetes. Br J Clin Pharmacol.

[7] American Diabetes Association (2019). Glycemic targets: standards of medical care in diabetes-2019. Diabetes Care.

[8] Pecoits-Filho R, Abensur H, Betônico CC (2016). Interactions between kidney disease and diabetes: dangerous liaisons. Diabetol Metab Syndr.

[9] Bonaventura A, Montecucco F, Dallegri F (2015). Update on strategies limiting iatrogenic Hypoglycaemia. Endocr Connect.

[10] Qian J, Scheer FAJL (2016). Circadian system and glucose metabolism: implications for physiology and disease. Trends Endocrinol Metab.

[11] Kant R, Yadav P, Kishore S, Kumar R, Bairwa M (2020). Circadian dysynchrony among nurses performing shift work at a tertiary care teaching hospital: a preliminary study. Int J Physiol Pathophysiol Pharmacol.

[12] Montaigne D, Marechal X, Modine T (2018). Daytime variation of perioperative myocardial injury in cardiac surgery and its prevention by Rev-Erbα antagonism: a single-center propensity-matched cohort study and a randomized study. Lancet.

[13] Qian J, Dalla Man C, Morris CJ, Cobelli C, Scheer FAJL (2018). Differential effects of the circadian system and circadian misalignment on insulin sensitivity and insulin secretion in humans. Diabetes Obes Metab.

[14] Unger T, Borghi C, Charchar F (2020). 2020 International Society of Hypertension global hypertension practice guidelines. J Hypertens.

[15] Blanc B, Finch CA, Hallberg L (1968). Nutritional anaemias. Report of a WHO Scientific Group. World Health Organ Tech Rep Ser.

[16] Nazar CM (2014). Diabetic nephropathy; principles of diagnosis and treatment of diabetic kidney disease. J Nephropharmacol.

[17] Petropoulos IN, Ponirakis G, Khan A (2018). Diagnosing Diabetic Neuropathy: Something Old, Something New. Diabetes Metab J.

[18] (2021). Centers for Disease Control and Prevention Coronary Artery Disease CDC.

[19] de Carlo TE, Chin AT, Bonini Filho MA (2015). Detection of microvascular changes in eyes of patients with diabetes but not clinical diabetic retinopathy using optical coherence tomography angiography. Retina.

[20] Kalra S, Mukherjee JJ, Venkataraman S (2013). Hypoglycemia: The neglected complication. Indian J Endocrinol Metab.

[21] Robinson RT, Harris ND, Ireland RH (2003). Mechanisms of abnormal cardiac repolarization during insulin-induced hypoglycaemia. Diabetes.

[22] Gerstein HC, Miller ME (2008). Effects of intensive glucose lowering in type 2 diabetes. N Engl J Med.

[23] Murad MH, Coto-Yglesias F, Wang AT (2009). Clinical review: Drug-induced Hypoglycaemia: a systematic review. J Clin Endocrinol Metab.

[24] Samya V, Shriraam V, Jasmine A (2019). Prevalence of hypoglycemia among patients with type 2 diabetes mellitus in a rural health center in South India. J Prim Care Community Health.

[25] AlKhaldi YM, AlKhaldi AY, AlQahtani AS (2019). Incidence of hypoglycemia and its risk factors among diabetics during Ramadan in Abha city, Aseer Region, KSA. J Family Med Prim Care.

[26] Kumar JG, Abhilash KP, Saya RP, Tadipaneni N, Bose JM (2017). A retrospective study on epidemiology of hypoglycemia in Emergency Department. Indian J Endocrinol Metab.

[27] Tsujimoto T, Yamamoto-Honda R, Kajio H (2015). Effectiveness of prior use of beta-blockers for preventing adverse influences of severe hypoglycemia in patients with diabetes: an observational study. Med (Baltimore).

[28] Amiel S, Sherwin R, Simonson D, Tamborlane WV (1988). Effect of intensive insulin therapy on glycemic thresholds for counterregulatory hormone release. Diabetes.

[29] Stanley S, Moheet A, Seaquist ER (2019). Central mechanisms of glucose sensing and counter regulation in defence of hypoglycaemia. Endocr Rev.

[30] Ulmer BJ, Kara A, Mariash CN (2015). Temporal occurrences and recurrence patterns of hypoglycemia during hospitalization. Endocr Pract.

[31] Yang C, Ma Y, Kang J (2015). Time and department distribution of hypoglycemia occurrences in hospitalized diabetic patients. Int J Nurs Sci.

[32] Ruan Y, Moysova Z, Tan G (2020). Inpatient hypoglycemia: understanding who is at risk. Diabetologia.

[33] Yun JS, Park YM, Han K (2019). Association between BMI and risk of severe hypoglycemia in type 2 diabetes. Diab Metab.

[34] Vilovic M, Kurir TT, Novak A (2020). Hypoglycemia and glucagon utilization in insulin-treated diabetic patients. Exp Clin Endocrinol Diab.

[35] Magee F, Bailey M, Pilcher DV (2018). Early glycemia and mortality in critically ill septic patients: Interaction with insulin-treated diabetes. J Crit Care.

[36] Park S, Kim DG, Suh GY (2012). Mild hypoglycemia is independently associated with increased risk of mortality in patients with sepsis: a 3-year retrospective observational study. Crit Care.

[37] Un JS, Ko SH (2016). Risk factors and adverse outcomes of severe hypoglycaemia in type 2 diabetes mellitus. Diabetes Metab J.

[38] Kim HM, Seong JM, Kim J (2016). Risk of hospitalization for Hypoglycaemia among older Korean people with diabetes mellitus: Interactions between treatment modalities and comorbidities. Medicine (Baltimore).

[39] Morales J, Schneider D (2014). Hypoglycemia. Am J Med.

[40] Costa Gil JE, Linari MA, Pugnaloni N (2017). Hypoglycaemia in patients with type 1 and type 2 diabetes mellitus on insulin therapy Results of the global HAT study in Argentina. Medicina (B Aires).

